# Shikonin Ameliorates LPS-Induced Cardiac Dysfunction by SIRT1-Dependent Inhibition of NLRP3 Inflammasome

**DOI:** 10.3389/fphys.2020.570441

**Published:** 2020-10-16

**Authors:** Tao Guo, Zhong-Biao Jiang, Zhong-Yi Tong, Yang Zhou, Xiang-Ping Chai, Xian-Zhong Xiao

**Affiliations:** ^1^Department of Emergency Medicine, Second Xiangya Hospital, Emergency Medicine and Difficult Diseases Institute, Central South University, Changsha, China; ^2^Department of Pathophysiology, Sepsis Translational Medicine Key Laboratory of Hunan Province, Xiangya School of Medicine, Central South University, Changsha, China; ^3^Department of Radiology, The Second Xiangya Hospital, Central South University, Changsha, China; ^4^Department of Pathology, The Second Xiangya Hospital, Central South University, Changsha, China

**Keywords:** shikonin, lipopolysaccharide, SIRT1, NLRP3, cardiac dysfunction

## Abstract

Shikonin (SHI) is an anti-inflammatory agent extracted from natural herbs. It is still unknown whether SHI ameliorates lipopolysaccharide (LPS)-induced cardiac dysfunction. This study aims to explore the protective effects of SHI on LPS-induced myocardial injury and its mechanism. The LPS-induced cardiac dysfunction mouse model was employed to investigate the protective effects of SHI. In the present study, we found that SHI treatment improved the survival rate and cardiac function and remarkably ameliorated the release of inflammatory cytokines and macrophage infiltration in heart tissue of LPS-treated mice. SHI also reduced lactate dehydrogenase (LDH) and cardiac troponin (cTn) release, cell inflammation, and apoptosis in LPS plus adenosine triphosphate (ATP)-treated H9c2 cells. In addition, SHI significantly upregulated silent information regulator 1 (SIRT1) expression and suppressed the upregulation of NOD-like receptor protein 3 (NLRP3), cleaved caspase-1, and caspase-1 activity in heart tissues induced by LPS. Meanwhile, we got the same results in LPS plus ATP-treated H9c2 cells *in vitro*. Further, SIRT1 inhibitor or siRNA partially blocked SHI-mediated upregulation of SIRT1 expression and downregulation of NLRP3, cleaved caspase-1, and caspase-1 activity in heart tissues induced by LPS. Therefore, we conclude that SHI ameliorates LPS-induced cardiac dysfunction by inhibiting SIRT1-dependent activation of NLRP3 inflammasomes and might be a promising therapeutic strategy for the treatment of LPS-induced cardiac dysfunction.

## Introduction

Cardiac dysfunction is a common complication of severe sepsis, which represents one of the leading causes of death in intensive care units (Court et al., 2002; Narvaez et al., 2018). Uncontrolled immune and inflammatory responses were involved in the main mechanisms of sepsis-induced cardiac dysfunction (SICD) (Kakihana et al., 2016; Liu et al., 2017). Unfortunately, even largely supportive therapies of SICD, like the clinical presence of antibiotics and restoration of systemic perfusion and blood pressure, were widely applied in the clinics, but the mortality of SICD was still high. The main reason is that the pathophysiological mechanisms of SICD are very complex. Therefore, further elucidation of the mechanisms is crucial for finding a new therapy for SICD.

The bacterial endotoxin lipopolysaccharide (LPS) was considered as the main culprit responsible for SICD (Bai et al., 2016). LPS stimulation resulted in the central inflammatory response and triggered rapid inflammasome activation, which included NOD-like receptor protein 3 (NLRP3), caspase-1, and apoptosis-associated speck-like protein (ASC). In these processes, inflammasome sensors recruited ASC to generate a caspase-1-activating platform and promote the maturation of pro-inflammatory cytokines, interleukin-1β (IL-1β) and IL-18 (Abderrazak et al., 2015; Karki and Kanneganti, 2019). Recently, many studies have confirmed that NLRP3 inflammasome was activated in sepsis-induced multiple organ injury. Furthermore, suppression of NLRP3 inflammasome activation ameliorated SICD (Kalbitz et al., 2016; Yang et al., 2018). Moreover, it has been reported that silent information regulator 1 (SIRT1), an NAD+-dependent deacetylase that regulates various protein functions, plays a negative role in regulating the activation of NLRP3 inflammasome (Fu et al., 2013; Peng et al., 2018). In addition, growing evidences demonstrated that SIRT1 played a protective role in SICD (Han et al., 2017; Smith et al., 2019). Therefore, activation of SIRT1-dependent pathways has become a promising therapeutic strategy for the treatment of SICD.

Shikonin (SHI) has antioxidant activity and anti-inflammatory properties, which is an active component extracted from Chinese herb radix arnebiae. It was reported that SHI could prevent multiple diseases, such as acute lung injury, lethal endotoxic shock, and cerebral ischemia/reperfusion injury (Wang et al., 2010; Andujar et al., 2013; Yang et al., 2014; Zhang Y. et al., 2018). Recent studies also demonstrated that SHI protected against cardiovascular diseases. For example, SHI ameliorated sympathetic remodeling in chronic heart failure (Liu and Liu, 2019). Additionally, SHI inhibited the activation of NLRP3 inflammasome in macrophages (Jin et al., 2015; Zorman et al., 2016). However, it is still unknown whether SHI could ameliorate LPS-induced cardiac dysfunction *via* an SIRT1-dependent NLRP3 inflammasome pathway. Hence, our study aims to assess the role of SHI in LPS-induced cardiac dysfunction and explore its underlying mechanisms.

## Materials and Methods

### Establishment of Lipopolysaccharide-Induced Cardiac Dysfunction Mouse Model

All animal-related experimental procedures were approved by the Animal Studies Ethics Committee of Central South University (Hunan, China). C57BL/6J male mice aged 6–8 weeks (20–24 g) were purchased from the Laboratory Animal Center of Central South University. LPS-induced cardiac dysfunction model was established in experimental mice by intraperitoneal injection of a dose 15 mg/kg of LPS (from *Escherichia coli* 0111:B4, Sigma Aldrich, St. Louis, MO, United States), which was dissolved in vehicle solution. The same volume of vehicle solution was given to the control group (Niu et al., 2011). Repetitive administration of SHI (#S7576, Sigma Aldrich, St. Louis, MO, United States) was given in each group at 0.5, 12, and 24 h after LPS challenge according to a previous study (Yang et al., 2014). The survival rates of mice were recorded every 6 h until 72 h after LPS challenge.

Mice were randomly divided into three groups as follows (*n* = 6 per group): (1) control group; (2) LPS group; (3) SHI+LPS group. The mice of the control group were injected intraperitoneally with vehicle solution. The LPS group established the model of SICD according to the method mentioned before. LPS+SHI group was injected intraperitoneally with SHI (8 mg/kg), which was dissolved in vehicle [10% dimethyl sulfoxide (DMSO), 20% cremophor:ethanol (3:1), and 70% phosphate-buffered saline] at 0.5 h before and 12 h after LPS challenge. Serum and heart tissues were collected after LPS challenge for 18 h and stored at −20°C for subsequent experiments.

### Echocardiography

Cardiac function was evaluated by echocardiography at 18 h after LPS challenge as previously described (Niu et al., 2011). Briefly, mice were lightly anesthetized and maintained on 0.5∼2.0% isoflurane, then they were placed and fixed in a recumbent position. Echocardiographic images were acquired by using Vevo 2000 imaging system (Toronto, Canada) equipped with a 30-mHz linear transducer (MS 400). Parameters of cardiac function were evaluated on the M-mode images, which were obtained from the parasternal short-axis view at the papillary muscle level. Left ventricle internal diameters at end-diastole and end-systole were determined. The ejection fraction (EF) and fractional shortening (FS) were analyzed to evaluate cardiac function. All data were blindly recorded by an independent investigator.

### Cell Culture and Lipopolysaccharide Induction

H9c2 cardiomyoblasts were bought from American Type Culture Collection and maintained in Dulbecco’s modified Eagle medium (DMEM) supplemented with 10% fetal bovine serum (FBS) and antibiotics (100 U/ml penicillin, 100 μg/ml streptomycin). Cells were grown on 12-well culture dishes until achieving 80∼90% confluence. The cells were divided into six groups as follows: control group, LPS+ATP group, LPS+ATP+EX527 group, SHI group, SHI+LPS+ATP group, and SHI+LPS+ATP+EX527 group. To examine the effects of SHI on inflammasome activation, cells were preincubated with SHI (5 mM) for 4 h at first, followed by priming with LPS (200 ng/ml) for another 4 h. After the replenishment of fresh medium, then pretreated with or without SIRT1 inhibitor EX527 (1 mM, Selleck Chemicals, Item No S1541) for 1 h immediately before stimulation with ATP. Finally, cells were stimulated with ATP (3 mM) for 1 h. Cells and supernatants were collected and stored at −80°C for further analysis.

### Cell Transfection

H9c2 cells were plated in six-well plates until 60–80% confluent. siRNA-negative control (NC) or siRNA-SIRT1 (50 nM) was diluted in Lipofectamine 2000 (Invitrogen, Carlsbad, CA, United States) and transfected into cells according to the manufacturer’s protocol. The sequences of SIRT1 siRNA were as follows: 5′-AGAUAUCAAUACAAUUGAAdTdT-3′ (F) and 5′-UUCAAUUGUAUUGAUAUCUdTdT-3′ (R). After 4 h, the medium was replaced with fresh DMEM containing 10% FBS, and cells were incubated for 48 h. Then, SHI was added to the fresh culture medium of cells for 4 h, and protein expression was measured by Western blot analysis.

### 3-(4,5-Dimethylthiazol-2-yl)-2,5-Diphenyl-Tetrazolium Bromide Assay

To assess cell viability, H9c2 cells were seeded into 96-well plates (Corning, NY, United States) and cultured at 37°C in the presence of 5% CO_2_ under a humidified atmosphere. At the indicated treatments, 20 μl 3-(4,5-dimethylthiazol-2-yl)-2,5-diphenyl-tetrazolium bromide (MTT) (5 mg/ml) was added into each well. After 4 h of incubation, cells were washed three times with NaCl/Pi (pH 7.4). The insoluble formazan product was dissolved in DMSO (150 μl/well). The absorbance was measured using a microplate reader at 570 nm with a 630-nm reference. The attendance of formazan formed in control cells was considered as 100% viability.

### Measurement of Creatine Kinase-MB, Lactate Dehydrogenase, Cardiac Troponin, Inflammatory Cytokines, and the Activity of Silent Information Regulator 1 and Caspase-1

The serum was collected and centrifuged at 3,000 rpm for 5 min. The levels of creatine kinase (CK) and CK-MB in serum were determined by Beckman LX-20 Fully Automated Biochemistry Analyzer (Beckman, California, United States) according to the manufacturer’s instructions. IL-1β ELISA kits (Cusabio Biotech Co., Ltd., Hubei, China; Item No. 011614MO) and IL-18 ELISA kits (Cusabio Biotech Co., Ltd., Hubei, China; Item No. E04609m) were used to determine IL-1β and IL-18 levels in serum and cell supernatants following manufacturer’s instructions. SIRT1 activity assay kit (Abcam, Cambridge, MA, United States; Item No. 156065) and caspase-1 activity assay kit (Beyotime, Nan Jing, China, Item No. C1101) were used to determine SIRT1 and caspase-1 activity in myocardial tissue or cell extracts.

### Hematoxylin and Eosin Staining

Mice were sacrificed, and heart tissues were dissected. Tissues from middle left ventricular were sectioned into 5-μm slices and stained with H&E staining solution according to the standard techniques. Histopathological changes were assessed by a light microscope at 200× magnification (Nikon, Tokyo, Japan). The cardiomyocyte cross-sectional areas were determined by testing the circumferential length of the cardiomyocyte using ImageJ software as described previously (Chen et al., 2018).

### Flow Cytometry Analysis

Cell apoptosis was assessed with Annexin V-FITC/PI Apoptosis Detection Kit (BD Biosciences, San Jose, CA, United States) and measured by flow cytometry. After stimulation, H9c2 cells were washed with cold PBS and stained with Annexin V-FITC and propidium iodide (PI) for 15 min at room temperature in the dark. Data were acquired by BD FACS flow cytometer (BD Bioscience, San Diego, CA, United States) and analyzed with Flow Jo analytical software (Version X; Tree Star, Ashland, OR, United States).

### Immunohistochemistry

The hearts were fixed in 10% formalin and embedded in paraffin. Embedded tissues were cut into 4-μm sections and incubated with macrophage marker F4/80 (1:50 dilution; CI-A3-1, Novus Biologicals, Centennial, CO, United States), followed by incubation with biotin-conjugated secondary antibodies and then treated with avidin peroxidase. The reaction was developed using the 3,3′-diaminobenzidine (DAB) substrate kit. Then, the sections were counterstained with hematoxylin and finally photographed under an optical microscope (Olympus, Tokyo, Japan) at 200× magnification. A semiquantitative evaluation of F4/80 was performed using a method described in the literature (Hu et al., 2013) as follows: the proportion of positive cells was divided into five grades (percentage scores): ≤10% = 0, 11–25% = 1, 26–50% = 2, 51–75% = 3, and >75% = 4. The intensity of staining was divided into four grades (intensity scores): no staining = 0, light brown = 1, brown = 2, and dark brown = 3. Staining positivity was determined by the formula as follows: Overall scores = Percentage score × Intensity score. All measurements were carried out in a double-blind manner by two independent researchers.

### Quantitative Real-Time Polymerase Chain Reaction

Total RNA was extracted from heart tissue and H9c2 cells by using TRIzol (Invitrogen, Carlsbad, CA, United States). The isolated RNA was used as a template to synthesize cDNA by using PrimeScript^TM^ RT Master Mix (Takara, Tokyo, Japan). Real-time PCR was carried out by using the One-step SYBR^®^. PrimeScript^TM^ RT-PCR Kit is a Biosystems 7500 instrument. The amplification conditions were 95°C for 30 s, 95°C for 5 s, and 60°C for 34 s for cycles. The specific primers were as follows: SIRT1, 5′-CCGTGGCAAACTGGTACTTT-3′ and 5′-G ACGCCAACATAGACCACCT-3′; β-actin, 5′-AAGTGTGAC GTTGACATCCG-3′ and 5′-TCTGCATCCTGTCAGCAATG-3′. Gene expression was normalized to β-actin expression using the software provided with the system.

### Western Blotting Analysis

The expressions of NLRP3, caspase-1, and SIRT1 in heart tissue and H9c2 cells at the protein level were determined by Western blotting analysis as previously described (Wang et al., 2017). Briefly, cardiac tissues and cells were minced and lysed in ice-cold radioimmunoprecipitation assay (RIPA) lysis buffer. Protein concentrations were determined by using the bicinchoninic acid (BCA) method. Equal amounts of proteins were subjected to sodium dodecyl sulfate polyacrylamide gel electrophoresis (SDS-PAGE) and then transferred to polyvinylidene fluoride (PVDF) membranes. The membranes were blocked with 5% milk for 2 h and then incubated with antibodies against SIRT1 (1:1,000 dilution, CST, Danvers, MA, United States; Item No. 8469S), NLRP3 (1:1,000 dilution, CST; Item No. 15101S), caspase-1 (1:1,000 dilution, CST; Item No. 3866S), and glyceraldehyde 3-phosphate dehydrogenase (GAPDH) (1:5,000 dilution, CST; Item No. 5174S) overnight. The membranes were washed three times, followed by incubation with fluorescence-conjugated secondary antibody at room temperature for 1 h. The immunoreactive bands were visualized by enhanced chemiluminescence (ECL) substrate (Bio-Rad, Hercules, CA, United States), and quantification was performed using ImageJ software (NIH, Bethesda, MD, United States).

### Statistical Analysis

SPSS 25.0 software was used for statistical analysis. All the values were expressed as means ± SD. A log-rank test was used to analyze survival curves. Comparisons between two groups were performed with a two-tailed Student’s *t*-test (parametric). Comparisons between multiple groups were analyzed using a one-way analysis of variance, followed by Tukey’s multiple comparison test. *P* < 0.05 was considered statistically significant.

## Results

### Shikonin Improves Survival and Ameliorates Cardiac Dysfunction in Lipopolysaccharide-Treated Mice

**FIGURE 1:**
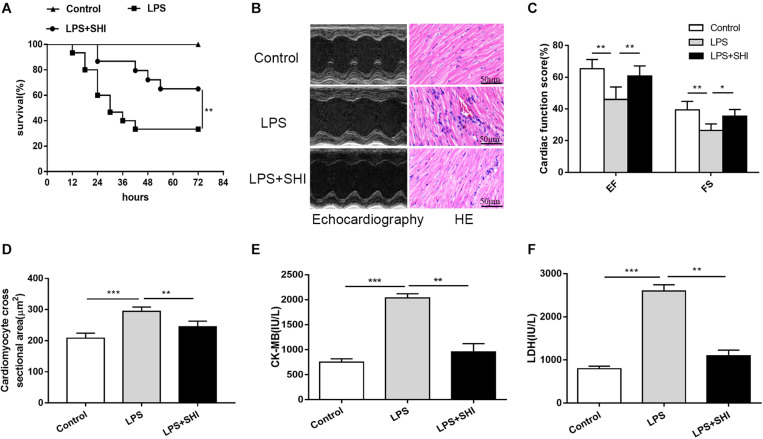
Shikonin (SHI) improves survival and ameliorates cardiac dysfunction in lipopolysaccharide (LPS)-treated mice. **(A)** Effect of SHI on survival rate following LPS challenge in mice (*n* = 15/group). **(B)** Effect of SHI on cardiac dysfunction and histomorphology after LPS treatment. **(C)** Cardiac function parameters, ejection fraction (EF) and fractional shortening (FS) were measured by echocardiography at 18 h after LPS treatment. **(D)** Cardiomyocyte cross-sectional areas were assessed in different groups. The levels of serum creatine kinase (CK)-MB **(E)** and lactate dehydrogenase (LDH) **(F)** were measured in different groups. All data were represented as mean ± SD (*n* = 6). *P < 0.05, **P < 0.01, ***P < 0.001.

It is still unknown whether SHI presents any protective efficacy on cardiac dysfunction induced by LPS. We firstly established a cardiac dysfunction mouse model induced by LPS and treated the mice with or without SHI. As shown in Figure 1A, the survival rate of mice in the LPS group significantly decreased to 26% compared to the control group. In contrast, treatment with 8 mg/kg SHI significantly improved the survival rate to 66%. Echocardiography evaluation also revealed that EF and FS were significantly reduced by LPS, while SHI treatment reversed the decreased effects on EF and FS induced by LPS (Figures 1B,C). Moreover, LPS markedly induced cardiac injury as evidenced by increasing serum levels of CK and LDH (Figures 1E,F). In contrast, SHI treatment attenuated the concentration of CK and LDH (Figures 1E,F). In addition, H&E staining of heart tissues showed that LPS increased the inflammatory cell infiltration, myocardial degeneration, and cardiomyocyte cross-sectional areas in heart tissues (Figures 1B,D). Conversely, SHI treatment significantly alleviated these pathological abnormalities of heart in LPS-challenged mice (Figures 1B,D). Thus, these data confirmed a protective effect of SHI against LPS-induced myocardial damage.

### Shikonin Reduces Inflammatory Cytokine Release and Macrophage Infiltration in the Heart Tissue of Lipopolysaccharide-Treated Mice

**FIGURE 2:**
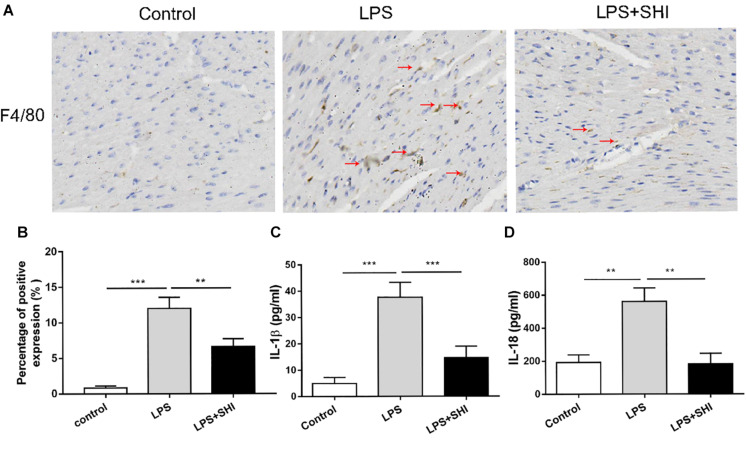
Shikonin (SHI) reduces inflammatory cytokine release and macrophage infiltration in heart tissue of lipopolysaccharide (LPS)-treated mice. **(A)** Immunohistochemistry assay was applied to detect macrophage infiltration (the arrow points to the macrophages). **(B)** Shows the quantification. Serum levels of interleukin (IL)-1β **(C)** and IL-18 **(D)** were determined with ELISA kits at 18 h after LPS treatment. All data were represented as mean ± SD (*n* = 6). *P < 0.05, **P < 0.01, ***P < 0.001.

Uncontrolled inflammatory responses were involved in SICD (Kakihana et al., 2016). To assess inflammatory response in SHI-treated mice, we measured the IL-1β and IL-18 in serum and macrophage infiltration in heart tissue. The results showed that macrophage infiltration in heart tissue (Figures 2A,B) and the levels of IL-1β and IL-18 in serum were significantly upregulated by LPS, while these effects were reversed by SHI (Figures 2C,D). These results confirmed that SHI could ameliorate inflammatory response of heart tissue in LPS-treated mice.

### Shikonin Alleviates Lactate Dehydrogenase Releases, Cell Inflammation, and Apoptosis in Lipopolysaccharide + ATP-Treated H9c2 Cells

**FIGURE 3:**
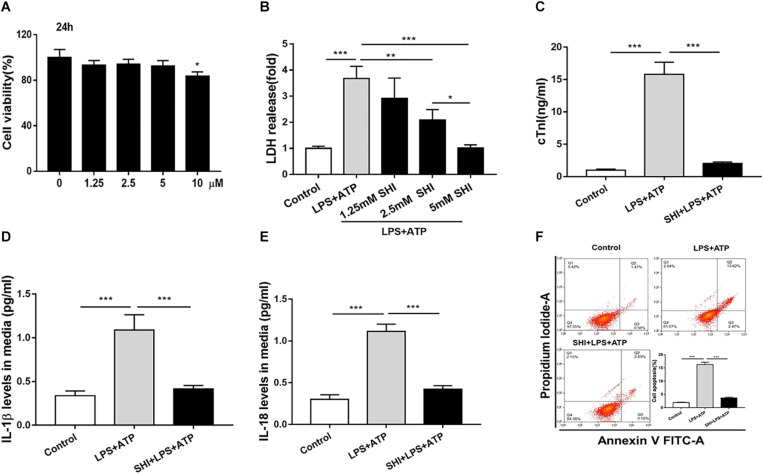
Shikonin (SHI) alleviates lactate dehydrogenase (LDH) releases, cell inflammation, and apoptosis in LPS + ATP-treated H9c2 cells. **(A)** 3-(4,5-Dimethylthiazol-2-yl)-2,5-diphenyl-tetrazolium bromide (MTT) assay was applied to examine cell viability in H9c2 cells that were treated with different concentrations of SHI for 24 h. **(B)** The effect of SHI on LDH release. **(C)** The effect of SHI on cardiac troponin (cTn) levels. The effect of SHI on interleukin (IL)-1β **(D)** and IL-18 **(E)** levels. **(F)** Cell apoptosis was assessed by flow cytometry. All data were represented as mean ± SD. All experiments were repeated at least three times. *P < 0.05, **P < 0.01. ***P < 0.001.

To investigate the protective role of SHI in LPS + ATP-treated H9c2 cells, cell viability, inflammatory cytokines, and cell apoptosis were determined. We found that SHI at doses of 1.25, 2.5, and 5 mM had no effect on cell viability of H9c2 cells compared to vehicle-treated cells, but at a dose of 10 mM decreased cell viability (Figure 3A), and SHI significantly reduced the LDH level in the supernatant of LPS + ATP-treated H9c2 cells at doses of 1.25, 2.5, and 5 mM, respectively, compared to PBS-treated cells (Figure 3B). The highest decrease in the levels of LDH was found to occur at a dose of 5 mM of SHI. Thus, we choose 5 mM of SHI as an optimal treatment concentration for the subsequent *in vitro* experiments. Besides, SHI reduced the cTn level in supernatants of LPS + ATP-treated H9c2 cells (Figure 3C). Additionally, ATP promoted the release of IL-1β and IL-18 and aggravated cell apoptosis in LPS-primed H9c2 cells, while SHI significantly reversed these effects induced by ATP in LPS-primed H9c2 cells (Figures 3D–F). Together, these results showed that SHI played a protective role in LPS + ATP-treated H9c2 cells.

### Shikonin Upregulates the Expression of Silent Information Regulator 1 and Inhibits the Activation of NOD-Like Receptor Protein 3 Inflammasome *In vivo* and *In vitro*

**FIGURE 4:**
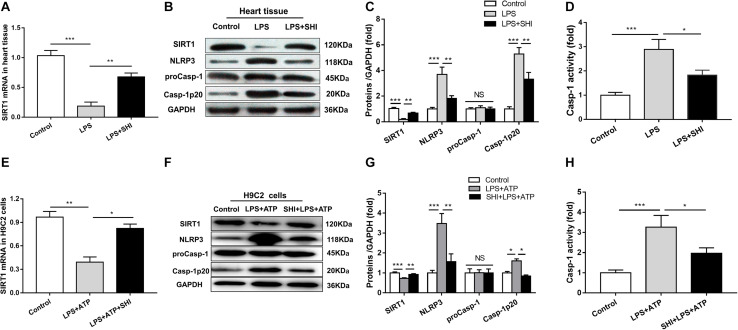
Shikonin (SHI) upregulates the expression of silent information regulator 1 (SIRT1) and inhibits activation of NOD-like receptor protein 3 (NLRP3) inflammasome *in vivo* and *in vitro*. **(A,E)** SIRT1 mRNA was measured by RT-PCR in heart tissue and H9c2 cells. **(B,F)** Expressions of SIRT1, NLRP3, pro-caspase-1, and cleaved caspase-1 were measured by Western blotting analysis in heart tissue and H9c2 cells. **(C,G)** The ratio of SIRT1, NLRP3, pro-caspase-1, cleaved caspase-1 to glyceraldehyde 3-phosphate dehydrogenase (GAPDH) was expressed as protein levels in heart tissue and H9c2 cells. **(D,H)** Caspase-1 activity was detected by a commercial kit in heart tissue and H9c2 cells. All data were represented as mean ± SD. All experiments were repeated at least three times. *P < 0.05, **P < 0.01, ***P < 0.001.

To investigate the mechanism of SHI’s protective effect on cardiac injury induced by LPS, the mRNA expression of SIRT1 was assessed by RT-PCR (Figures 1A,E), the protein expressions of SIRT1, NLRP3, pro-caspase-1, and cleaved caspase-1 in the heart tissues and H9c2 cells were determined by Western blotting analysis (Figures 4B,F), and caspase-1 activity was measured by a commercial kit. We found that the mRNA and protein expressions of SIRT1 in heart tissues were significantly decreased by LPS, while SHI significantly upregulated mRNA and protein levels of SIRT1 in heart tissues of the LPS group (Figure 4A). Meanwhile, the protein expressions of NLRP3 and cleaved caspase-1 and caspase-1 activity in heart tissues were significantly upregulated by LPS, while SHI treatment significantly suppressed the upregulation of NLRP3, cleaved caspase-1 (Figures 4B,C), and caspase-1 activity (Figure 4D) induced by LPS. In addition, we got the same results in H9c2 cells *in vitro*. ATP downregulated the mRNA and protein expressions of SIRT1 in LPS-primed H9c2 cells, while SHI upregulated the mRNA and protein levels of SIRT1 in LPS-primed H9c2 cells stimulated with ATP (Figure 4E). The protein expressions of NLRP3 and cleaved caspase-1 and caspase-1 activity were enhanced by ATP in LPS-primed H9c2 cells, while SHI treatment significantly decreased the upregulation of NLRP3, cleaved caspase-1 (Figures 4F,G), and caspase-1 activity (Figure 4H) induced by LPS-primed H9c2 cells. These findings indicated that SHI upregulated SIRT1 expression and inhibited the activation of NLRP3 inflammasome *in vivo* and *in vitro*.

### Silent Information Regulator 1 Inhibitor Prevents the Inhibitory Effect of Shikonin on Inflammation in Lipopolysaccharide + ATP-Treated H9c2 Cells

**FIGURE 5:**
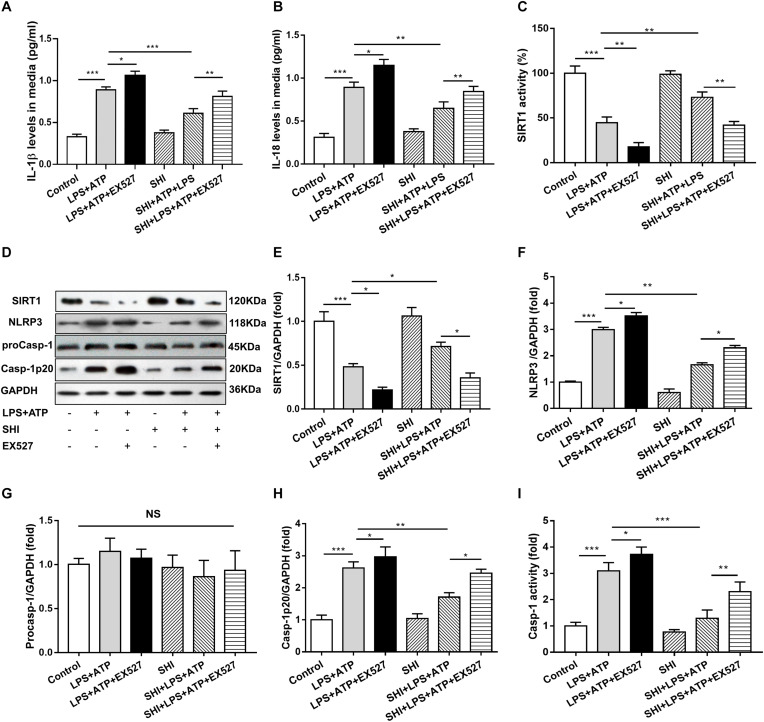
Silent information regulator 1 (SIRT1) inhibitor prevents the inhibitory effect of shikonin (SHI) on inflammation in lipopolysaccharide (LPS) + ATP-treated H9c2 cells. The levels of interleukin (IL)-18 **(A)** and IL-1β **(B)** in the supernatant of LPS + ATP-treated H9c2 cells were determined by ELISA. **(C)** SIRT1 activity in H9c2 cells was measured by a commercial kit. **(D)** Expressions of SIRT1, NOD-like receptor protein 3 (NLRP3), pro-caspase-1, and cleaved caspase-1 protein in H9c2 cells were measured by Western blotting analysis. The ratio of SIRT1 **(E)**, NLRP3 **(F)**, pro-caspase-1 **(G)**, or cleaved caspase-1 **(H)** to glyceraldehyde 3-phosphate dehydrogenase (GAPDH) was expressed at protein levels in H9c2 cells. **(I)** Caspase-1 activity was detected by a commercial kit in H9c2 cells. All data were represented as mean ± SD. All experiments were repeated at least three times. *P < 0.05, **P < 0.01, ***P < 0.001.

To further confirm whether SHI inhibits the release of inflammatory cytokines through SIRT1 pathway in LPS + ATP-treated H9c2 cells, SHI and SIRT1 inhibitor (EX527) were used. Results indicated that both of the protein expression and activity of SIRT1 were decreased in LPS-primed H9c2 cells stimulated with ATP, while SHI restored the reduction in protein expression and activity of SIRT1. The protein expressions of NLRP3 and cleaved caspase-1 and caspase-1 activity were upregulated in LPS + ATP-treated H9c2 cells. In contrast, SHI inhibited the protein levels of NLRP3 and cleaved caspase-1 (Figures 5C–H) and caspase-1 activity (Figure 5I) in LPS + ATP-treated H9c2 cells. Moreover, EX527 prevented such inhibitory effects of SHI on the activation of NLRP3 inflammasome. In addition, the production of IL-1β and IL-18 in cell supernatants was increased in LPS + ATP-treated H9c2 cells compared to the control group, which was attenuated by SHI. Nevertheless, EX527 could block SHI-mediated inhibitory effects of pro-inflammatory cytokine release (Figures 5A,B). These data indicated that the SIRT1 inhibitor prevented the inhibitory effects of SHI on activation of NLRP3 inflammasome inflammatory factor release in LPS + ATP-treated H9c2 cells.

### Silent Information Regulator 1 Knockdown Suppresses Shikonin-Mediated Anti-inflammatory Activity

**FIGURE 6:**
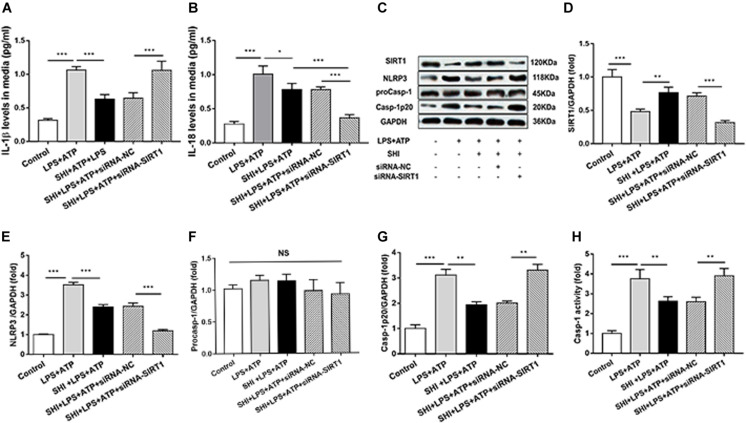
Silent information regulator 1 (SIRT1) knockdown suppresses shikonin (SHI)-mediated anti-inflammatory activity. The levels of interleukin (IL)-18 **(A)** and IL-1β **(B)** in the supernatant of lipopolysaccharide (LPS) + ATP-treated H9c2 cells were determined by ELISA. **(C)** Expressions of SIRT1, NOD-like receptor protein 3 (NLRP3), pro-caspase-1, and cleaved caspase-1 protein in H9c2 cells were measured by Western blotting analysis. The ratio of SIRT1 **(D)**, NLRP3 **(E)**, pro-caspase-1 **(F)**, or cleaved caspase-1 **(G)** to glyceraldehyde 3-phosphate dehydrogenase (GAPDH) was expressed at protein levels in H9c2 cells. Caspase-1 activity **(H)** was detected by a commercial kit in H9c2 cells. All data were represented as mean ± SD. All experiments were repeated at least three times. *P < 0.05, **P < 0.01, ***P < 0.001.

To provide solid evidence that SIRT1 participates in the anti-inflammatory effects of SHI in H9c2 cells, siRNA-SIRT1 were used to knock down SIRT1 in the present study. The result showed that the expression of SIRT1 was decreased to 44% by siRNA transfection, which indicates that siRNA-SIRT1 transfection was effective (Figures 6C,D). The protein levels of NLRP3 and cleaved caspase-1 (Figures 6E–G) and caspase-1 activity (Figure 6H) were upregulated by ATP in LPS-primed H9c2 cells, while these effects were reversed by treatment with SHI. Moreover, siRNA-SIRT1 suppresses SHI-mediated production of IL-1β and IL-18 and NLRP3 inflammasome activation (Figures 6A–G). The IL-1β and IL-18 secretions in cell supernatants were increased by ATP in LPS-primed H9c2 cells compared to the control group, which was reduced by treatment with SHI. Furthermore, siRNA-SIRT1 partially abolished SHI-mediated reduction of IL-1β and IL-18 secretion (Figures 6A,B). These results indicated that knockdown of SIRT1 by siRNA could suppress SHI-mediated anti-inflammatory activity in LPS + ATP-challenged H9c2 cells.

## Discussion

**FIGURE 7:**
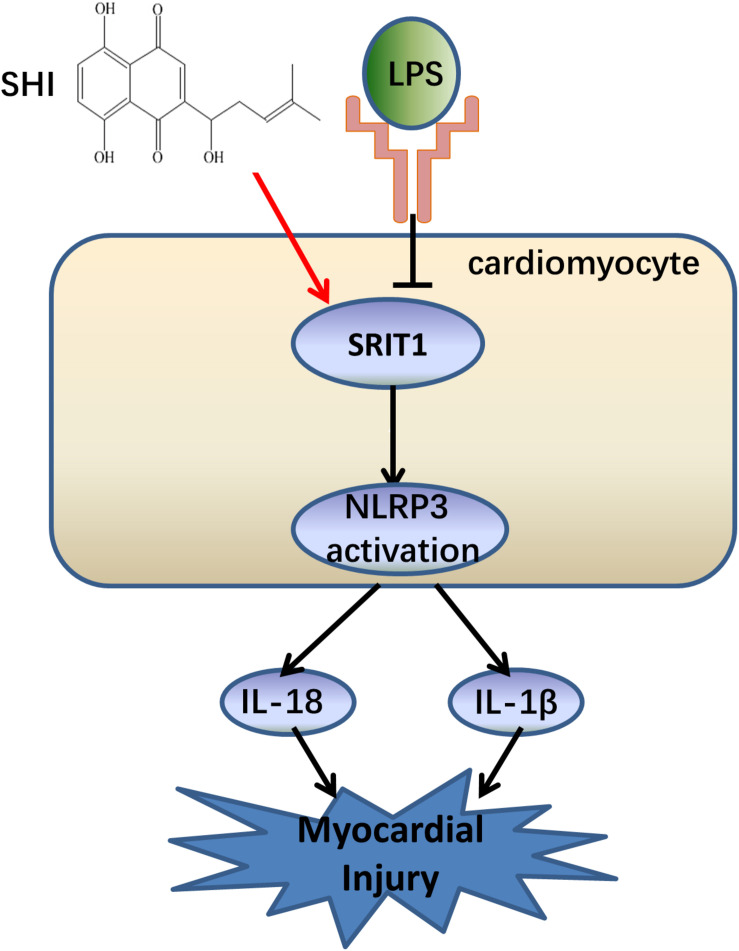
The summary flow diagram.

In the present study, we confirmed that NLRP3 inflammasome activation significantly contributed to the pathogenesis of LPS-induced cardiac dysfunction, which was consistent with a previous study (Yang et al., 2019). Meanwhile, the study was the first time to demonstrate that SHI has a protective effect on LPS-induced cardiac dysfunction. Furthermore, SHI significantly reduced cell inflammation by inhibiting the activation of NLRP3 inflammasome *in vivo* and *in vitro*. Mechanistically, SHI ameliorated LPS-induced cardiac dysfunction by inhibiting the activation of NLRP3 inflammasome that was partly mediated by SIRT1 in mice (Figure 7).

SHI has various biological functions, and it is well known due to its effective anti-inflammatory activity. Many studies have shown that SHI is beneficial for many diseases, such as cerebral ischemia- or reperfusion-induced brain injury and sepsis (Wang et al., 2010; Yang et al., 2014). Importantly, Yang et al. (2017) reported that SHI ameliorated isoproterenol-induced myocardial injury. Moreover, some reports recently confirmed that SHI could block high-mobility group box protein 1 (HMGB1) release in activated macrophages. However, the relationship between SHI and LPS-induced cardiac dysfunction has not been studied. Thus, we established a LPS-induced cardiac dysfunction mouse model and found that SHI could reverse cardiac function, restore the destruction of myocardial architecture, reduce serum cardiac enzymes and inflammation response, and elevate survival rate. In addition, we found that SHI could alleviate LDH releases, pro-inflammatory cytokine release (IL-1β and IL-18), and cell apoptosis in LPS-treated H9c2 cells. Some evidences confirmed that IL-18 could induce tumor necrosis factor (TNF)-α, IL-1β, IL-6, and nitric oxide (NO) production, which resulted in cardiac myocyte apoptosis (Keira et al., 2002). Additionally, IL-18 induces proapoptotic Fas, Fas-L, and TNFR1 expression in endothelial cells (Marino and Cardier, 2003). Thus, SHI suppression of ATP-induced cell apoptosis in LPS-primed H9c2 cells is an indirect effect by inhibiting pro-inflammatory cytokine production. Therefore, these results indicated that SHI could play a protective role in LPS-induced cardiac injury.

The activation of the NLRP3 inflammasome requires two steps: priming and activation. The priming step is that pattern recognition receptors (signal 1) such as Toll-like receptors activate nuclear factor-κB (NF-κB) pathways, leading to the formation of the NLRP3–ASC–caspase-1 complex (Swanson et al., 2019). Then, stimulation of damage-associated molecular patterns (signal 2, such as ATP and nigericin) provokes the activation of the NLRP3 inflammasome, mediating the maturation and secretion of IL-1β and IL18 (Mangan et al., 2018; Swanson et al., 2019). Cumulative evidences showed that the activation of NLRP3 inflammasome played a key role in sepsis-induced myocardial injury (Zhang et al., 2015; Wu et al., 2018). Yang et al. (2018) also found that the expression levels of NLRP3 and caspase-1 were increased in myocardial tissue treated with cecal ligation and puncture. Consistent with these results, we also found that the expressions of NLRP3 and cleaved caspase-1 at the protein level in heart tissues and caspase-1 activity were increased by LPS. Additionally, we found that SHI significantly reduced the production of IL-1β and IL-18 and macrophage infiltration of heart tissue in LPS-treated mice, which may be related to the activation of NLRP3 inflammasome. Additionally, we also found that SHI significantly reduced the increased production of IL-1β and IL-18 in cell culture supernatants and also inhibited the activation of NLRP3 inflammasome and enhanced caspase-1 activity by ATP in LPS-primed H9c2 cells. Thus, these data indicated that the protective effect of SHI on the cardiac injury was due to inhibiting the activation of NLRP3 inflammasome and inflammatory cytokine release.

SIRT1, a 3-hydroxy-3-methylglutaryl coenzyme A (HMG-CoA) reductase, is a key regulator in various intracellular processes. A recent study reported that mRNA and protein expression of SIRT1 was increased in septic cardiomyopathy, and treatment with EX527 (a selective SIRT1 inhibitor) could improve cardiac function such as increased global longitudinal strain and longitudinal strain rate (Smith et al., 2019). Notably, SIRT1 has been considered to be involved in the inflammatory response of macrophages cells (Jia et al., 2018). In the present study, we observed that SHI increased SIRT1 protein levels in heart tissue and H9c2 cells, which may be accountable for its upregulation of SIRT1 mRNA levels. However, some evidences reported that SHI inhibited mRNA and protein expressions of SIRT1 in HepG2 cells with overexpression of SIRT1 (Jin et al., 2015). These differences may be contributed to different regulatory effects of SHI on SIRT1 in different cell models. We further examined whether SIRT1 was involved in the inhibitory effects of SHI in NLRP3 inflammasome activation in H9c2 cells. Here, we found that LPS-induced reduction of SIRT1 activity and protein levels as well as the activation of NLRP3 inflammasome were prevented by SHI. Intriguingly, EX-527 not only inhibited SIRT1 activity but also downregulated SIRT1 at the protein levels. These results were consistent with recent reports (Smith et al., 2019). However, it was previously reported that EX527 only inhibited SIRT1 catalytic activity (Napper et al., 2005; Solomon et al., 2006). The reason may be that after EX527 binds to the active area of SIRT1, it blocks the inhibition of downstream inflammatory factors. These inflammatory cytokines could decrease the protein expression of SIRT1 through positive feedback. In addition, EX527 abolished the inhibitory effects of SHI on the activation of NLRP3 inflammasome, SIRT1 knockdown suppresses SHI-mediated anti-inflammatory activity. The probable mechanisms of SIRT1-induced inhibition of the activation of NLRP3 inflammasome were that SIRT1 deacetylated and blocked some transcriptional factors, such as Kruppel-like factor 4 (KLF4) and NF-κB, and then further decreased the expression of NLRP3 inflammasome-associated protein (Franceschelli et al., 2016; Zhang X. et al., 2018).

## Conclusion

In summary, our findings provided the first evidence that SHI exerted a protective role against LPS-induced cardiac dysfunction. The protective effect of SHI was attributed to the inhibition of NLRP3 inflammasome activation mediated by SIRT1 *in vivo* and *in vitro*. Consequently, our data demonstrated that SHI is a promising therapeutic target for SICD.

## Data Availability Statement

All datasets presented in this study are included in the article/Supplementary Material.

## Ethics Statement

All animal-related experimental procedures were approved by Animal Studies Ethics Committee of Central South University (Hunan, China). Written informed consent was obtained from the owners for the participation of their animals in this study.

## Author Contributions

TG, Z-BJ, X-PC, and X-ZX conceived and designed the experiments. TG, Z-BJ, and Z-YT executed the experiments and analyzed the samples. TG, Z-BJ, and Z-YT analyzed the data. TG wrote the first version of the manuscript. All authors interpreted the data, critically revised the manuscript, and approved the final version of the manuscript.

## Conflict of Interest

The authors declare that the research was conducted in the absence of any commercial or financial relationships that could be construed as a potential conflict of interest.
